# Quality of life of older persons in nursing homes after the implementation of a knowledge‐based palliative care intervention

**DOI:** 10.1111/opn.12258

**Published:** 2019-07-12

**Authors:** Christina Bökberg, Lina Behm, Gerd Ahlström

**Affiliations:** ^1^ Department of Health Sciences, Faculty of Medicine Lund University Lund Sweden

**Keywords:** ageing, education, nursing homes, palliative care, quality of life

## Abstract

**Background:**

The goals of palliative care are to relieve suffering and promote quality of life. Palliative care for older persons has been less prioritised than palliative care for younger people with cancer, which may lead to unnecessary suffering and decreased quality of life at the final stage of life.

**Aim:**

To evaluate whether a palliative care intervention had any influence on the perceived quality of life of older persons (≥65 years).

**Methods:**

This study was conducted as a complex intervention performed with an experimental crossover design. The intervention was implemented in 20 nursing homes, with a six‐month intervention period in each nursing home. Twenty‐three older persons (≥65 years) in the intervention group and 29 in the control group were interviewed using the WHOQOL‐BREF and WHOQOL‐OLD questionnaires at both baseline and follow‐up. The collected data were analysed using the Wilcoxon signed‐rank test to compare paired data between baseline and follow‐up.

**Results:**

In the intervention group, no statistically significant increases in quality of life were found. This result contrasted with the control group, which revealed statistically significant declines in quality of life at both the dimension and item levels. Accordingly, this study showed a trend of decreased health after nine months in both the intervention and control groups.

**Conclusion:**

It is reasonable to believe that quality of life decreases with age as part of the natural course of the ageing process. However, it seems that the palliative care approach of the intervention prevented unnecessary quality of life decline by supporting sensory abilities, autonomy and social participation among older persons in nursing homes. From the ageing perspective, it may not be realistic to strive for an increased quality of life in older people living in nursing homes; maybe the goal should be to delay or prevent reduced quality of life. Based on this perspective, the intervention prevented decline in quality of life in nursing home residents.

**Implications for practice:**

The high number of deaths shows the importance to identify palliative care needs in older persons at an early stage to prevent or delay deterioration of quality of life.

**What does this research add to the existing knowledge in gerontology?**

- It seems that the palliative care approach in this intervention prevented unnecessary QoL decline by supporting sensory abilities, autonomy and social participation among older persons living in nursing homes.
- Frail older persons are often excluded from research; this study is one of few intervention studies on palliative care in nursing homes reporting outcomes of the residents.
- There is an urgent need to continue testing different models to identify effective implementation strategies for palliative care due to the increased number of older persons in many countries.

**What are the implications of this new knowledge for nursing care with older people?**

- This intervention model reduces the gap in the limited knowledge regarding how palliative care can be implemented in nursing homes.
- The high number of deaths shows that it is important that staff are able to identify older persons in nursing homes who are in need of palliative care in the early stage.
- Due to the progression of multi‐morbidity in frail older persons living in nursing homes, a realistic QoL goal is to delay or prevent its deterioration.

**How could the findings be used to influence policy, practice, research or education?**

- This intervention recommends that staff and manager seminars are held more frequently than every five weeks.
- The dropout rate due to death or insufficient energy was considerably high, so a narrower follow‐up period is recommended when frail older persons are included in experimental studies.
- Education regarding evidence‐based high‐quality palliative care is a promising model that can be performed in nursing homes; therefore, this palliative care approach can be easily disseminated to all staff.

## 1 INTRODUCTION

The palliative care approach is used to promote quality of life (QoL), reduce symptoms and relieve suffering in persons with progressive, life‐threatening illnesses or injuries, despite the age, diagnosis and context of care (Connor & Sepulveda Bermedo, 2014; Davies & Higginson, 2004; Hall, Petkova, Tsouros, Constantini, & Higginson, 2011; Sawatzky et al., 2016). Regardless of the facts that 38% of all deaths in Sweden occur in nursing homes (Håkansson, Öhlén, Morin, & Cohen, 2015) and 72% of all people who died in Sweden in 2017 were 75 years or older (Statistics Sweden, 2018), older persons dying of multiple morbidities or “old age” at nursing homes receive insufficient palliative care. This insufficient care may lead to older persons experiencing unnecessary suffering (Fleming et al., 2017; Smedbäck et al., 2017) and decreased QoL at the final stage of life.

Older people have been less prioritised in terms of palliative care compare to younger people with cancer. However, older persons are more likely to die from other chronic diseases, often several progressive diseases, implying complex care needs (Davies & Higginson, 2004; Froggatt et al., 2017). The most common causes of death of older persons in Sweden are heart and circulatory diseases, cancer and dementia (Smedbäck et al., 2017; Statistics Sweden, 2018). These diseases usually progress over a period of time, which makes it challenging to determine when an older person's end‐of‐life stage begins (Goddard, Stewart, Thompson, & Hall, 2013); therefore, early signs preceding death could be difficult for the nursing home staff to detect (Åvik, Sandgren, Fürst, Ahlström, & Behm, 2018). The end‐of‐life process of older persons has been described as a dynamic individual process (Krishnan, 2017), and the final year of life is often associated with symptoms such as pain, depression, confusion and distress (Fleming et al., 2017), leading to decreased QoL. A high proportion of older people die shortly after moving into a nursing home (Schon, Lagergren, & Karleholt, 2016), and almost one‐third of older persons who move into a nursing home die within six weeks (Smedbäck et al., 2017). This situation implies that most of the persons living in nursing homes are in the final stage of life and would benefit from a palliative care approach.

Today, QoL is considered a crucial outcome in healthcare research, and the underlying reason to measure QoL in health care is to ensure that the evaluations focus on the person rather than the disease (Leegaard et al., 2018). To understand QoL in old age, not only do the distresses and impairments resulting from poor health need to be considered but non‐health‐related aspects must be considered as well (Lawton, 1999). Even if there is no consensus regarding the concept of QoL, it must address the individuals’ perceptions of both positive and negative dimensions (Bowling et al., 2015; World Health Organization, 1995, 1997) and include different domains (emotional, physical, social, independence, spirituality and environmental) of a person's well‐being (World Health Organization, 1995, 1997). These domains capture the important ways in which healthcare conditions impact a person's QoL and can bring about a meaningful understanding to improve the final stage of life.

A growing number of older people will require palliative care in nursing home settings (Froggatt et al., 2017; Gott & Ingleton, 2011; Murtagh et al., 2014). However, previous research has noted the need for improved palliative care for older people in these settings (De Gendt, Bilsen, Vander, & Deliens, 2013; Gott & Ingleton, 2011; Schon et al., 2016). Not only are symptom and suffering relief lacking (Fleming et al., 2017; Smedbäck et al., 2017) but the possibilities for older persons to share their thoughts of life and death or talk about passing away with the staff are also insufficient (Österlind, Ternestedt, Hansebo, & Hellström, 2017). Additionally, requests from staff, that is., nurse assistants, for more education on palliative care as well as for support from Registered Nurses and managers have been described earlier (Beck, Törnquist, Broström, & Edberg, 2012). A study of 322 nursing homes in six European countries found that knowledge concerning basic palliative care was suboptimal in all participating countries (Smets et al., 2018). With to the aim of improving palliative care and thereby the QoL of older persons in nursing homes, a Swedish research group implemented a knowledge‐based palliative care intervention directed at staff and managers in 20 nursing homes in Sweden (Ahlström et al., 2018). This study aimed to evaluate whether the palliative care intervention had any influence on the perceived QoL of older persons (≥65 years).

## 2 METHODS

### 2.1 Research setting and design

This study is a part of the “KUnskapsbaserad PAlliativ vård” [in English: “Knowledge‐based Palliative Care”] project, which is abbreviated as the KUPA project. The KUPA project was conducted from 2015 to 2017 as a complex intervention performed with an experimental crossover design (Ahlström et al., 2018). The KUPA project is designed from an implementation perspective, described in the previous study protocol for the project (Ahlström et al., 2018). In this research approach, a successful change in practice depends on the interplay between several aspects: (a) effectiveness of the implementation strategies; (b) characteristics of the “implementation object”; (c) characteristics of the implementers; (d) the target population; and (e) the context of the implementation (Ahlström et al., 2018; Damschroder et al., 2009; Nilsen, 2015). Therefore, the evaluation of the whole KUPA project addresses several different perspectives which will be reported in future papers; staff, managers, context (Alftberg et al., 2018) the older persons and their next of kin and also various barriers and facilitating aspects of the implementation (Ahlström et al., 2018).

The knowledge‐based palliative care intervention was implemented with various staff members (Registered Nurses, assistant nurses, social workers, occupational therapists and physiotherapists) and front‐line leaders over a 6‐month period in 20 nursing homes in two different counties in the south of Sweden (County A and County B). Firstly, the intervention was implemented in ten nursing homes in County A, while ten nursing homes in County B served as a control group. Then, the ten control nursing homes in County B implemented the intervention, and ten new nursing homes in County A, which had not received the intervention, were chosen as a control group (Ahlström et al., 2018).

### 2.2 The knowledge‐based palliative care intervention

The intervention consisted of five 2‐hr seminars and was based on two Swedish national documents describing the key principles of palliative care: a care programme designed by the Regional Co‐operative Cancer Centres (2012) and a knowledge‐based document created by the National Board of Health and Welfare (2013). Both documents were created with the intent to improve the quality of the palliative care provided and are based on the World Health Organization (WHO) definition of palliative care (Connor & Sepulveda Bermedo, 2014; Davies & Higginson, 2004; Hall et al., 2011). The focuses on the seminars were (a) the palliative approach and dignified care, (b) next of kin, (c) existence and dying, (d) symptom relief and (e) collaborative care. For further details of the intervention and the seminars, please see Ahlström et al. (2018) and Bökberg, Behm, Wallerstedt & Ahlström (2019).

### 2.3 Sampling and participants

The nursing homes in this study were selected through voluntary participation. This resulted in a mixture of larger and smaller nursing homes from both urban and rural areas (Ahlström et al., 2018).

The inclusion criteria for the older persons were that they were 65 years or older, spoke Swedish, did not have dementia and had enough energy to participate in a structured interview for up to one hour. The recruitment of the older persons was conducted consecutively in equal numbers from both the intervention nursing homes and the control nursing homes. In total, 90 older persons were included in the study. Thirty‐eight (42%) dropouts were related to death, not enough energy, and/or lack of interest in participating in the follow‐up interview. Altogether, 23 older persons in the intervention group and 29 in the control group were interviewed at both baseline and follow‐up and were included in this study. The demographic variables of the two groups were similar, with a median age of 87 years, and most of the included older persons were female, widowers or widows (Table 1).

**Table 1:** Characteristics of the older persons in the intervention and control groups at baseline

Demographic variable	Intervention group (*n* 23)	Control group (*n* 29)
Age		
Median (range), years	87 (73–101)	87 (66–98)
Gender (*n*/%)		
Female	16 (70)	16 (55)
Marital status (*n*/%)		
Married	6/26	7/24
Widower/Widow	13/57	18/62
Divorced	1/4	2/6.5
Single	3/13	2/6.5

### 2.4 Data collection

A contact person was designated at each of the included nursing homes and informed the older persons who met the inclusion criteria about the aim and design of the study. If the older person responded positively regarding participation in the study, their oral informed consent was requested. Then, the contact person passed the personal data of the older persons to the researchers, and the time and place for the interview were determined. Before the interview, the information about the study was repeated, and the older person had the opportunity to ask questions before signing the written informed consent. With the intention of obtaining reliable data, the older persons answered the two questionnaires in the form of a structured interview. The four interviewers who asked the older persons the questions from the questionnaires were all Registered Nurses and had experience interviewing older persons. The response options were printed in large text on a separate paper to assist the older persons in answering the questions (Ahlström et al., 2018).

### 2.5 Instruments

The data collection was based on two questionnaires that are commonly used together to assess QoL in older persons (Power, Quinn, & Schmidt, 2005), the WHOQOL‐BREF and the WHOQOL‐OLD. The questionnaires are based on the WHO definition of QOL as a broad, multidimensional concept of an individual's perception of his or her position in life in the context of the culture and value systems in which a person lives and in relation to his or her personal goals, expectations, standards and concerns (World Health Organization, 1995). Both questionnaires were answered before (baseline) and three months after (follow‐up) the intervention was completed, that is, nine months from baseline. The same time intervals applied to the questionnaires in the control nursing homes.

*The WHOQOL‐BREF* consists of a total of 26 questions that assess individual facets related to QoL, with the exception of two general questions: one related to “the overall quality of life” and one about “general health.” The instrument consists of four domains: (a) *physical health*, containing seven items; (b) *psychological,* containing six items; (c) *social relationships,* containing three items; and (d) *environment,* containing eight items. The older persons were asked to respond on a five‐point positively rated Likert scale. The total score of the scale ranges from 26 to 130, with higher values indicating a better quality of life (World Health Organization, 1996). The psychometric properties of the WHOQOL‐BREF items have demonstrated high test–retest reliability and validity in older adults (Chachamovich, Trentini, & Fleck, 2007; Steinbüchel, Lischetzke, Gurny, & Eid, 2006). Due to the Regional Ethics Review Board in Lund (reference number: 2015/4), the item “sexual activity” in the *social relationships’* domain needed to be removed. The WHO gave permission to exclude the item to (GA), the project leader of KUPA. Thus, the total score in this study ranged from 25 to 125. 0.66, 0.73, 0.38, 0.60.

*The WHOQOL‐OLD questionnaire* consists of a total of 24 questions and is used to assess QoL in older adults. The questionnaire consists of six domains containing four items each: (a) *sensory abilities;* (b) *autonomy;* (c) *past, present and future abilities;* (d) *social participation;* (e) *death and dying;* and (f) *intimacy*. Respondents are asked to respond on a five‐point positively rated Likert scale. The total score of the scale ranges from 24 to 120, with higher values indicating a better perceived QoL (World Health Organization, 2006). The psychometric properties of the WHOQOL‐OLD in older adults have been validated in several countries and have demonstrated high test–retest reliability and validity (Power et al., 2005) 0.79, 0.50, 0.59, 0.79, 0.80, 0.63.

### 2.6 Statistical analyses

The quantitative data collected from questionnaires were analysed using within‐group comparisons (the data from after the intervention period were compared with the data from before the intervention period in the intervention group and the control group). The selection of the statistical tests to analyse data other than the descriptive statistics was based on whether the data were distributed normally and the scale level of the instruments. A Wilcoxon signed‐rank test was used to compare paired data from after the intervention period to the data from before the intervention period. Subgroup analysis, using the Mann–Whitney U test and the Kruskal–Wallis test, was applied to compare baseline characteristics of the intervention group and the control group. The same analyses were applied for the dropouts in the intervention group with the participants in the intervention group and the dropouts within the control group with the participants in the control group. Either Pearson's chi‐square test or Fisher's exact test was used when the expected value was <5. Analyses were performed using IBM SPSS Statistics version 24. A two‐tailed *P*‐value of ≤0.05 was regarded as statistically significant. Missing data for single items were replaced by the mean score for that item (World Health Organization, 1996, 2006).

### 2.7 Ethical considerations

The Regional Ethics Review Board in Lund approved the KUPA project (reference number: 2015/4). The project is also registered under trial registration NCT02708498. The ethical research principles for medical research guided this study in accordance with the ethical standards of the Declaration of Helsinki (World Medical Association, 2013). The study was conducted in agreement with the Swedish Ethical Review of Research Involving Humans Act (SFS, 2003:460), the General Data Protection Regulation [GDPR] (European Data Protection Board, 2018) and the Public Access and Secrecy Law (SFS, 2009:400).

To enable the older persons to make an autonomous decision regarding whether or not to participate in the study, both verbal information and written information about the study were provided to the older persons prior to the interviews. Before the written consent was signed, the older persons were informed that participating in the interviews was voluntary and that they could interrupt at any time without having to give a reason and without any consequences. The interviews were conducted by researchers, that is, Registered Nurses trained for the task and with experience approaching older persons to minimise risks of inflicting harm. The principle of non‐maleficence was maintained since the older persons were guaranteed confidentiality; that is, individuals cannot be identified as the older person's personal data were encoded. Only the codes were used during the data analysis, and the findings are reported on the group level. The code lists are stored away from the questionnaire forms, which are in locked cabinets. To create as beneficial of an environment as possible for the older persons, the older persons themselves chose the time and place for the interviews and breaks were taken if the older person got tired.

## 3 RESULTS

No statistically significant differences were detected when comparing baseline characteristics (age, gender, marital status, overall QoL and general health) of the intervention and control groups as well as the dropouts in the intervention group with the participants in the intervention group and the dropouts within the control group with the participants in the control group. The total median score from the QOL‐BREF questionnaire for the intervention group at baseline was 84 (first quartile (Q1)‐third quartile (Q3) = 77–97). The corresponding score for the control group was 85 (Q1–Q3 = 77–92.5). Three months after the knowledge‐based palliative care intervention was completed, a non‐significant increase was reported in the intervention group, with a median score of 86 (Q1–Q3 = 74–95), while the control group reported a non‐significant decrease, with a median score of 82 (Q1–Q3 = 77.5–90) (Table 2).

**Table 2:** Intervention group (*n* = 23) and control group (*n* = 29) analyses of the WHOQOL‐BREF before and after the implementation of the intervention

Domains and items	Intervention group Baseline Median (Q1–Q3)	Intervention group Follow‐up Median (Q1–Q3)	*p*‐value^b^	Control group Baseline Median (Q1–Q3)	Control group Follow‐up Median (Q1–Q3)	*p*‐value^b^
**Total score** (25–125)^a^	84 (77–97)	86 (74–95)	0.322	85 (77–92.5)	82 (77.5–90)	0.094
**General QoL** (1–5)^a^	3 (3–4)	3 (2–4)	0.477	3 (3–4)	3 (2.5–4)	0.073
**General health** (1–5)^a^	4 (2–4)	3 (2–4)	0.377	4 (2–4)	3 (2.5–4)	0.869
**Physical health** (7–35)^a^ Activities of daily living Dependence on medicinal substances and medical aids Energy and fatigue Mobility Pain and discomfort Sleep and rest Work capacity	21 (17–24)	21 (18–25)	0.651	20 (19–25)	20 (17–24.5)	0.390
**Psychological** (6–30)^a^ Bodily image and appearance Negative feelings Positive feelings Self‐esteem Spirituality/religion/personal beliefs Thinking, learning memory and concentration	21 (18–24) 5 (3–5) 3 (3–4)	21 (20–23) 3 (3–5) 3 (2–4)	0.896 0.200 0.285	21 (18–24) 3 (3–4.5) 3 (3–4)	20 (18–21.5) 3 (3–4) 3 (2–3)	0.062 **0.047** ^**4**^ **0.013** ^**3**^
**Social relationships** (2–10)^a^ Personal relationships Social support	8 (7–9)	8 (7–8)	0.495	8 (7–8)	8 (7.5–9)	0.239
**Environment** (8–40)^a^ Financial resources Freedom, physical safety, and security Health and social care: accessibility and quality Home environment Information Participation and opportunities for recreation/leisure activities Physical environment (pollution/noise/traffic/climate) Transportation	31 (28–33)	28 (25–33)	0.196	30 (26.5–33)	29 (26–31)	0.173

Note

Q1 = first quartile; Q3 = third quartile. Significant values are given in bold.

a Underlined score is the most favourable score.

b Wilcoxon Signed Rank Test.

The median score for “general QoL” was three in both groups at baseline, with no statistically significant change at follow‐up. The median score for “general health” was four in both groups at baseline, with a non‐significant decrease to a median score of three at follow‐up. It should be noted that both groups reported a decline in the domain of *Environment* even though the change was not statistically significant (Table 2).

When comparing the data at the item level, the results revealed that the control group demonstrated a statistically significant decline in the *psychological* dimension for both “bodily image and appearance” (*p*‐value 0.047) and “negative feelings” (*p*‐value 0.013) (Table 2). The questions for those items were “Are you able to accept your bodily appearance?” and “How often do you have negative feelings such as sadness, despair, anxiety, depression?” No further item‐level results from the QOL‐BREF are presented since the analyses did not reveal any statistically significant changes.

When comparing the results from the QOL‐OLD questionnaire, statistically significant decreases at both the dimension and item levels were revealed in the control group. The median score in the *sensory abilities* dimension decreased from 16 to 13 (*p*‐value 0.001), the median score in the *autonomy* dimension decreased from 15 to 12 (*p*‐value 0.000), and the median score in the *social participation* dimension decreased from 15 to 13 (*p*‐value 0.000). In the intervention group, no statistically significant changes were found for any of the dimensions (Table 3).

**Table 3:** Intervention group (*n* = 23) and control group (*n* = 29) analyses of the WHOQOL‐OLD before and after the implementation of the intervention

Domains and items	Intervention group Baseline Median (Q1–Q3)	Intervention group Follow‐up Median (Q1–Q3)	*p*‐value^b^	Control group Baseline Median (Q1–Q3)	Control group Follow‐up Median (Q1–Q3)	*p*‐value^b^
**Sensory abilities (5–20)** a	15 (11–17)	15 (12–17)	0.378	16 (12–20)	13 (11–15.5)	**0.001**
Impairments to senses that affect daily life						
Loss of sensory abilities that affect participation in activities				3 (2–4)	4 (3–5)	**0.005**
Problems with sensory functioning that affect the ability to interact						
Rate sensory functioning (1–5)^a^						
**Autonomy (5–20)** a	12 (11–14)	11 (10–15)	0.115	15 (14–17)	12 (10–13)	**0.000**
Freedom to make own decisions						
Feel in control of your future						
People around you are respectful of your freedom						
Able to do things you'd like (1–5)^a^	3 (2–4)	3 (2–4)	0.731	3 (2–3)	2 (2–3)	**0.019**
**Past, present and future abilities (5–20)** a	14 (11–15)	14 (11–15)	0.072	14 (12–15)	14 (12–15)	1.000
Satisfied with opportunities to continue achieving						
Received the recognition you deserve in life (1–5)^a^				4 (3–5)	3 (3–4)	**0.040**
Satisfied with what you've achieved in life (1–5)^a^						
Happy with things to look forward to						
**Social participation (5–20)** a Have enough to do each day Satisfied with the way you use your time Satisfied with level of activity Satisfied with opportunities to participate in community	13 (10–14)	12 (9–16)	0.349	15 (12–18)	13 (10–14)	**0.000**
**Death and dying (5–20)** a Concerned about the way you will die Afraid of not being able to control death Scared of dying Fear pain before death	18 (15–20)	17 (14–19)	1.000	14 (15.5–20)	18 (15–19)	0.939
**Intimacy (5–20)** a Feel a sense of companionship in life Experience love in your life Opportunities to love Opportunities to be loved	13 (11–16)	14 (12–16)	0.896	13 (12–16)	14 (12–16)	0.896

Note

Q1 = first quartile; Q3 = third quartile. Significant values are given in bold.

a Underlined score is the most favourable score.

b Wilcoxon Signed Rank Test.

In the control group, statistically significant changes were found for three items. The item “able to do things you like” decreased from a median score of 3 at baseline to a median score of 2 at follow‐up (*p*‐value 0.019) and the item “received the recognition you deserve in life” decreased from a median score of 4 at baseline to a median score of 3 at follow‐up (*p*‐value 0.040). Furthermore, an increase was found in the control group for “loss of sensory abilities that affect participation in activities” from a median score of 3 to a median score of 4 (*p*‐value 0.005) (Table 3). No further item‐level results from the QOL‐OLD are presented since the analyses did not reveal any other statistically significant changes.

## 4 DISCUSSION

This intervention study aimed to evaluate whether a palliative care intervention had any influence on the perceived quality of life of older persons. We found no statistically significant increases in QoL of the older persons in the intervention group; however, we found statistically significant declines in QoL in the control group at both the dimension and item levels. This result implies that, unlike the control group, the intervention group did not experience reduced QoL. It is reasonable to believe that QoL decreases with age, as the health and functional status of very old persons are expected to deteriorate over time in the natural course of the ageing process (Schon et al., 2016). Accordingly, this study also showed a similar trend of decreased health after nine months in both the intervention and control groups, which could explain the absence of increased QoL. From the perspective of ageing, it may not be realistic to strive for an improved QoL in frail older people such as those living in nursing homes; instead, maybe the goal should be to delay or prevent decreased QoL. Based on this perspective, the intervention prevented a decline in QoL in nursing home residents.

The palliative care approach aims to improve the quality of life for persons facing problems associated with the end of life (Hall et al., 2011; Sawatzky et al., 2016). However, palliative care intervention studies involving residents in nursing homes are sparse. One Cochrane review published in 2011 found few studies to include, and few outcomes for residents were assessed (Hall et al., 2011). Results from this study show that the control group exhibited decreased QoL in the dimensions of *sensory abilities*, *autonomy* and *social participation*. Sensory abilities are a perceived consequence of sensory impairments that, according to the Global Burden of Disease project (World Health Organization, 2015), were identified as the greatest burden of disability for ageing people throughout the world. More than 180 million older people are estimated to have hearing loss that hinders normal conversational speech. Additionally, complex impairments in the eyes have important implications for the everyday lives of older people (World Health Organization, 2015). If the staff neglect to identify hearing or/and vision losses in frail older people at a nursing home, these impairments will be untreated, which negatively affects physical activities and communication and contributes to social isolation and loss of autonomy (Evans & Rowlands, 2004; Weinstein, 2015; World Health Organization, 2015). This may explain the significant decrease in this study regarding *autonomy* and *social participation* observed in the control group. A contradictory result in the control group was the significant improvement in the loss of *sensory abilities* affect participation in activities, which indicates that staff recognise these sensory impairments among the older people. Annual health checks for older people in nursing homes may prevent adverse hearing and visual impairments.

In the *autonomy* domain, the three scores decreased in the control group, while the scores of the intervention group were stable over time. This result is supported by a qualitative study, where a total of 50 staffs were interviewed about autonomy at nursing homes in Sweden. These researchers found that the residents were labelled by the staff as old people who were unable and unwilling to strive for autonomy (Wikström & Emilsson, 2014). Furthermore, a study regarding QoL in Irish nursing homes showed that the more dependent the resident was on the staff, the less likely they were to have a choice (Murphy, Shea, & Cooney, 2007). This fact might explain the significant decrease in this study over time in the items “able to do things you like” in the *autonomy* dimension and received the recognition you deserve in life in the *past, present and future abilities* dimension. For the older persons to do the things they like to do, the staff needs to get to know the preferences of the residents. The palliative care intervention in this study promoted the use of advanced care planning (ACP) for all nursing home residents. ACP is a discussion with the resident or a representative of the resident about the goals and desired direction of the patient's care. One RCT study using ACP for 309 older persons aged 80 and over found that when using ACP, end‐of‐life wishes were much more likely to be known (Detering, Hancock, Reade, & Silvester, 2010). Furthermore, a systematic review of 113 studies using ACP in different settings (Brinkman‐Stoppelenburg, Rietjens, & van der Heide, 2014) found evidence that ACP positively impacts the quality of care at the end of life.

Earlier evaluations of the care of older persons in nursing homes have shown that the care can often be task‐centred and does not correspond to the individual's needs (Beck et al., 2012). By introducing a palliative care approach in which the older person is seen in a holistic view (World Health Organization, 2006), the staff might be open to the idea that the care should be individualised according to each resident's need. A qualitative study by Reitinger, Schuchter, Heimerl, and Wegleitner (2018) exploring relatives’ views of the palliative care culture in nursing homes found that the change in culture is highly dependent on management. The relatives concluded that in order to create a palliative care culture, management must support the approach and develop the appropriate structures. However, an qualitative study of managers in the KUPA project (Nilsen, Wallerstedt, Behm, & Ahlström, 2018) showed that the managers experience limited organisational readiness to develop evidence‐based palliative care as a result of variation in the nursing home staff's change efficacy and change commitment as well as restrictions related to many contextual conditions.

### 4.1 Methodological considerations

Frail older persons are often excluded from research (Lloyd‐Williams, Kennedy, Sixsmith, & Sixsmith, 2007; Smedbäck et al., 2017; Ternestedt & Franklin, 2006; Towsley, Hirschman, & Madden, 2015), and this study is one of few intervention studies on palliative care in nursing homes reporting outcomes for residents. However, this study poses some methodological problems that will be discussed below.

Firstly, despite including 30 nursing homes and 90 older persons in this study, it was difficult to recruit participants for the interviews who had enough energy and who had stable mental and physical health over time. Consistent with earlier research (Björk et al., 2016; Hoffmann, Kaduszkiewicz, Glaeske, Bussche, & Koller, 2014), most of the residents had a degree of mental impairment or were too physically frail to participate. Despite this challenge, we succeeded with our goal; however, the frail nature of the participants induced a challenge. The dropout rates related to death and not having enough energy were considerable (42%) and negatively affected the statistical power of the study. Therefore, after nine months from baseline, the sample of participants became small (*n* = 52), which also decreased the generalisation of the results, even though the recruiting procedure was carried out in both small and large nursing homes as well as in both urban and rural areas. It should be noted that no statistically significant differences regarding background variables were detected between the dropouts and the included older persons. Including a larger number of nursing homes was not justifiable from a resource point of view but poses a threat to the effectiveness of the intervention. In fact, the residents in nursing homes represent the frailest older population in Sweden, with a median survival time of six to nine months (National Board of Health & Welfare, 2018). The voice of the older persons themselves is crucial for palliative care research.

Furthermore, we encountered some problems related to the data collection. The questions from the WHOQOL‐BREF and WHOQOL‐OLD were asked by an interviewer who filled in the answers. Despite this, we noticed that the older persons had difficulties understanding the response options, which resulted in some missing answers. One action that we took to make the response options easier to understand was to enlarge the answers and to ask the question several times. Furthermore, the interviewers were experienced nurses with extensive experience conversing with older persons. The fact that it was difficult to understand the questions and response options could have affected the reliability of the results. This difficulty also affected the interview time. The interviews lasted for 45 min to 2 hr, which was tiring for some of the participants. If the interviews lasted longer than one hour, a break or a chance to continue the interview another day was offered. This was a way to respect the frail condition of the older participant and maintain the principle of non‐maleficence.

QoL can be measured by a variety of instruments. The World Health Organization (2006) recommends the use of the WHOQOL instruments in a wide variety of contexts, which makes the results highly valid. The instruments have both been validated in several countries and been demonstrated high test–retest reliability and validity (Chachamovich et al., 2007; Power et al., 2005; Steinbüchel et al., 2006). However, one question in the WHOQOL‐BREF questionnaire was excluded for ethical reasons based on a decision from the Ethical board, which lowers the validity of the *social relationships’* domain.

## 5 CONCLUSIONS

The evaluation showed no increase in the QoL dimensions, which could be an expected result of a six‐month educational palliative care intervention. The intervention group exhibited no decline in the QoL dimensions at follow‐up. However, the control group showed significant declines in the sensory abilities, autonomy and social participation dimensions. It seems that the palliative care approach in the intervention in this study prevented unnecessary reductions in QoL by supporting sensory abilities, autonomy and social participation among frail older persons living in nursing homes. However, it is important to continue developing research‐based knowledge about effective palliative care interventions as the proportion of frail older persons increases worldwide. From the perspective of reaching enough statistical power, future multicentre studies are recommended. Future research needs to test and evaluate educational models including increasing the frequency of staff training and extending the period beyond six months before measuring if any improvements occurred of QOL for the older persons living in nursing homes.

6

1 **Implications for practice**

- The high number of deaths shows the importance to identify palliative care needs in older persons at an early stage to prevent or delay deterioration of quality of life.

## CONFLICT OF INTEREST

The authors declare that they have no conflict of interests.

## AUTHORS CONTRIBUTION

Analysis of the data and drafting the article: CB; The project leader, GA, design of the study, national research grant and recruiting process: GA (the project leader); interviews with the older persons: LB. All authors contributed to the content of the manuscript text and critically reviewed, discussed, and approved the final version of the manuscript.

## Data Availability

The data sets analysed in the current study are not publicly available due to sensitive information from a very vulnerable group, namely frail older persons. Even though the data were anonymised, the study contains sufficient details to enable the identification of individuals. Therefore, before approving the study, the Regional Ethics Review Board in Lund set severe restrictions regarding the accessibility of the data, but the data are available from the project leader (GA) on reasonable request.
